# The impact of donor type on the outcome of pediatric patients with very high risk acute lymphoblastic leukemia. A study of the ALL SCT 2003 BFM-SG and 2007-BFM-International SG

**DOI:** 10.1038/s41409-020-01014-x

**Published:** 2020-08-04

**Authors:** Jean-Hugues Dalle, Adriana Balduzzi, Peter Bader, Anna Pieczonka, Isaac Yaniv, Arjan Lankester, Marc Bierings, Akif Yesilipek, Petr Sedlacek, Marianne Ifversen, Peter Svec, Jacek Toporski, Taifun Gungor, Jacek Wachowiak, Evgenia Glogova, Ulrike Poetschger, Christina Peters

**Affiliations:** 1 Department of Pediatric Hemato-Immunology, Hôpital Robert Debré, APHP Nord—Université de Paris, Paris, France; 2 Clinica Pediatrica, Università degli Studi di Milano Bicocca, Fondazione MBBM/Ospedale San Gerardo, Monza, Italy; 3 Division for Stem Cell Transplantation and Immunology, Department for Children and Adolescents, University Hospital Frankfurt, Goethe University, Frankfurt am Main, Germany; 4 Poznan University of Medical Sciences, Depratment of Pediatric Oncology, Hematology and HSCT, Poznan, Poland; 5 The Raina Zaizov Pediatric Hematology Oncology Division, Schneider Children’s Medical Center of Israel, Petach Tikva, Israel; 6 Willem-Alexander Children’s Hospital, Leiden University Medical Center, Leiden, the Netherlands; 7 Department of Hematology, University Hospital of Children, Utrecht, the Netherlands; 8 Pediatric Stem Cell Transplantation Unit, Medical Park Antalya Hospital, Antalya, Turkey; 9 Department of Paediatric Haematology and Oncology, University Hospital Motol, Prague, Czech Republic; 10 Rigshospitalet, Paediatric Clinic II, Copenhagen, Denmark; 11 Department of Paediatric Haematology and Oncology, Haematopoietic Stem Cell Transplantation Unit, Comenius University Children’s Hospital, Bratislava, Slovakia; 12 Department of Hematology, Skanes University Hopsital, Lund, Sweden; 13 Pediatric Stem Cell Transplantation, UZH, University of Zurich, Zurich, Switzerland; 14 St Anna Children’s Hospital, Universitätsklinik für Kinder- und Jugendheilkunde, Medizinische Universität Wien, Vienna, Austria

**Keywords:** Acute lymphocytic leukaemia, Risk factors

## Abstract

Allogeneic HSCT represents the only potentially curative treatment for very high risk (VHR) ALL. Two consecutive international prospective studies, ALL-SCT-(I)BFM 2003 and 2007 were conducted in 1150 pediatric patients. 569 presented with VHR disease leading to any kind of HSCT. All patients >2 year old were transplanted after TBI-based MAC. The median follow-up was 5 years. 463 patients were transplanted from matched donor (MD) and 106 from mismatched donor (MMD). 214 were in CR1. Stem cell source was unmanipulated BM for 330 patients, unmanipulated PBSC for 135, ex vivo T-cell depleted PBSC for 62 and cord-blood for 26. There were more advanced disease, more ex vivo T-cell depletion, and more chemotherapy based conditioning regimen for patients transplanted from MMD as compared to those transplanted from MSD or MD. Median follow up (reversed Kaplan Meier estimator) was 4.99 years, median follow up of survivals was 4.88, range (0.01–11.72) years. The 4-year CI of extensive cGvHD was 13 ± 2% and 17 ± 4% (*p* = NS) for the patients transplanted from MD and MMD, respectively. 4-year EFS was statistically better for patients transplanted from MD (60 ± 2% vs. 42 ± 5%, *p* < 0.001) for the whole cohort. This difference does not exist if considering separately patients treated in the most recent study. There was no difference in 4-year CI of relapse. The 4-year NRM was lower for patients transplanted from MD (9 ± 1% vs. 23 ± 4%, *p* < 0.001). In multivariate analysis, donor-type appears as a negative risk-factor for OS, EFS, and NRM. This paper demonstrates the impact of donor type on overall results of allogeneic stem cell transplantation for very-high risk pediatric acute lymphoblastic leukemia with worse results when using MMD stem cell source.

## Introduction

For decades, allogeneic hematopoietic stem cell transplantation (HSCT) was developed as an additional treatment for patients presenting with high risk malignant hematological diseases and may represent the only curative option for very high risk acute lymphoblastic leukemia. The achievement of complete remission (CR) through conventional chemotherapy ± local radiotherapy (on central nervous system—CNS) appeared as a key or as an absolute pre-requisite for the procedure success before applying HSCT. Many different parameters play a role into the final results measured by both overall survival (OS), event-free survival (EFS), non relapse mortality (NRM) and more recently defined graft-versus-host-relapse-free-survival such as disease profile risk at diagnosis, disease status at the time of HSCT (CR1/CR2 vs beyond CR2), donor type (sibling, full-HLA compatible donor, haplo-donor), stem cell source (Bone Marrow (BM), Peripheral Blood Stem Cell (PBMC), Cord Blood Unit (CBU)), conditioning regimen (TBI-based, chemo-based, myelo-ablative conditioning regimen vs. reduced toxicity conditioning regimen vs. less intensive conditioning regimen i.e., non myéloablative conditioning regimen and reduced intensity ones). The definition of risk of disease recurrence is changing over time [1–4]. Currently it’s mainly based on biological and molecular characteristics at time of diagnosis and on minimal residual disease (MRD) level after one or two courses of chemotherapy.

Our international consortium conducted two successive prospective studies, namely BFM ALL-SCT 2003 and International BFM ALL-SCT 2007. A total of 1115 consecutive patients were enrolled using the identical criteria for defining the relapse risk (Standard vs. High Relapse Risk (HRR) vs. Very High Relapse Risk VHRR)) and the donor type (Matched Sibling (MSD) vs. Matched Donor (MD) vs. Mismatched Donor (MMD)).

Different previously published papers reported the main outcome for patients transplanted from either MSD or MD within BFM ALL SCT 2003 study [5] and International-BFM ALL SCT 2007 [6], as well as for patients transplanted from alternative donor in both studies [7]. Papers from Peters et al. and Balduzzi et al. demonstrated the non-inferiority of transplantation from a “9 or 10 out of 10” allelic matched donor, either related or unrelated, over the transplantation from an HLA-identical sibling in terms of EFS, OS, and CIR. In our hands, being transplanted from HLA-identical siblings versus “9 or 10 out of 10” matched donor had no significant impact on final outcome including death and relapse in the multivariate analyses. Paper from Dalle et al. reported that results obtained after HSCT from mismatched donor are acceptable but remained inferior to those obtained from better matched stem cell source for patients with very-high-risk of relapse ALL. Even though NRM was higher, compared with fully matched graft recipients, relapse remained the major cause of treatment failure also in MMD recipients.

The last part of these two studies is the report of the sub-analysis about the outcome of the 569 VHRR-patients depending of the donor-type. This is the purpose of the current paper.

## Patients and methods

Patients and methods are already published in the previous papers and then are provided here as Supplementary data (CS1: Patients and Methods and Tables S1A and S1B) [5–7].

Both studies were prospective, multicenter open trials (extended as a register studies until 2013), approved by the central and local ethical committees. Informed consents were obtained from parents or legal guardians and assent from patients, when appropriate, prior to study entry. International-ALL SCT 2007 was registered under Eudract 2005-005106-23.

### Statistical analysis

For non-time to event variables, Chi-Square tests, or where appropriate Fisher’s exact test, were used to compare groups for categorical variables, and the Wilcoxon rank-sum test (Kruskal–Wallis test for more than two populations) was used for continuous variables. The OS and EFS probabilities were calculated using the Kaplan–Meier method, and the groups were compared using the log-rank test. For OS, death resulting from any cause was defined as an event, and for EFS, the events included relapse, secondary malignancy, and death of any cause. The starting point for survival analysis was the date of the first HSCT. Survivors were censored at the last follow-up.

The cumulative incidence of chronic GVHD, relapse (CIR) and non-relapse mortality (NRM, defining as death in remission), were estimated using the Kalbfleisch and Prentice approach [8], accounting for competing risks, which included death in remission and relapse for GVHD, death in remission for CIR, relapse for NRM, and secondary malignancy for all measures of outcome described above. Comparisons were made according to the Gray test [9].

For multivariable analyses, we used logistic regression to model the impact of risk factors on the incidence of aGVHD (data not censored until 100 days after HSCT). The impact of prognostic factors on EFS and OS was evaluated using the Cox proportional hazards model, and the impact on chronic GVHD, CIR and NRM were evaluated using the Proportional Subdistribution Hazards model of Fine and Gray for censored data subject to competing risks [10]. The variables included into the multivariate models were patient, disease and donor characteristics, known to impact on outcomes (i.e., age of recipient and donor, gender match, remission status at time of SCT, disease cytomegalovirus (CMV) serostatus, phenotype, stem cell source and conditioning regimen). All *p* values below 0.05 were considered significant. The statistical analysis was performed using SAS version 9.4 (SAS Institute, Cary, NC).

All transplanted patients were assessed for general analyses, but only patients transplanted for VHRR disease were reported here.

The median follow-up was 5 years.

## Results

Between 2003 and 2013, 1115 patients, up to the age of 18 years, presenting with ALL in first or subsequent complete remission (CR) were enrolled in either the ALL SCT 2003 BFM Study (*n* = 705) or the ALL SCT 2007 International BFM Study (*n* = 410). Among these patients, 569 underwent HSCT for VHRR, 368 were enrolled in the 2003 BFM study, and the 201 remaining patients were enrolled in the international protocol.

Median follow up (reversed Kaplan Meier estimator) was 4.99 years, median follow up of survivals was 4.88, range (0.01–11.72) years.

The patient characteristics are shown in Table 1. As both previous papers demonstrated the non-inferiority between patients transplanted from MSD and those transplanted from MD, these two patient groups have been merged for the purpose of this analysis. Briefly, 463 patients were transplanted from either MSD or MD and 106 patients from MMD. There were 377 males and 192 females (gender ratio: 1.96). A total of 407 patients presented with B-cell lineage ALL, and 127 (106 and 21 transplanted from MSD/MD and MMD, respectively) with T lineage ALL and 19 (15 and 4) patients suffered from either Ph-positive or MLL-AF4 ALL, respectively. The median age at HSCT was 10.2 years (range: 0.9–23.1), and 277 patients below the age of 10 years were transplanted. A total of 214 patients were transplanted in CR1, 270 patients in CR2, and 85 patients in CR > 2. Four hundred and forty-eight patients received a TBI-based conditioning regimen. One-hundred and forty-one male recipients were transplanted from a female donor. The stem cell sources were ex vivo T-cell depleted PBSCs for 62 patients (either by positive CD34^+^ selection or by negative CD3^+^/CD19^+^ depletion), unmanipulated PBSCs for 135 patients, unmanipulated bone marrow for 330 patients, and cord-blood for 26 patients. One hundred and thirteen CMV serological-positive patients received transplants from CMV serological-negative donors. GvHD prophylaxis was performed according to protocol for patients transplanted with unmanipulated grafts.

**Table 1:** Patient characteristics.

		Patients (total)		MSD + MD		MMD		*p* value
Total	*N*	569		463		106		
Age of patient at SCT	Median (range)	10.2 (0.9–23.1)		10.3 (0.9–23.1)		8.9 (1.0–20.6)		0.130
≤4 years	*N*	69	12%	52	11%	17	16%	0.315
>4 and ≤12 years	*N*	293	51%	238	51%	55	52%	
>12 years	*N*	207	36%	173	37%	34	34%	
Age of donor								
≤18 years	*N*	107	21%	105	25%	2	2%	<0.001
>18 and ≤35 years	*N*	205	41%	182	43%	23	28%	
>35 years	*N*	191	38%	135	32%	56	69%	
Patients with stem cell source CB	*N*	26		9		17		
Missing	*N*	40		32		8		
Gender donor/patient								
Donor-female, patient-male	*N*	141	25%	117	26%	24	24%	0.638
Others	*N*	413	75%	335	74%	78	76%	
Missing	*N*	15		11		4		
Remission status at SCT								
CR1	*N*	214	38%	178	38%	36	36%	0.022
CR2	*N*	270	47%	225	49%	45	42%	
CR > 2	*N*	85	15%	60	13%	25	24%	
Phenotype of patient								
b-cell	*N*	407	74%	330	73%	77	77%	0.243
t-cell	*N*	127	23%	106	23%	21	21%	
Other	*N*	18	3%	16	4%	2	2%	
Not available	*N*	17		11		6		
Graft source/manipulation								
BM unmanipulated	*N*	330	60%	310	69%	20	20%	<0.001
PB unmanipulated	*N*	135	24%	118	26%	17	17%	
CB unmanipulated	*N*	26	5%	9	2%	17	17%	
Ex vivo manip. PB	*N*	62	11%	15	3%	47	47%	
Graft manipulation data missing	*N*	16		11		5		
CMV status donor/patient								
Donor-negative, patient-positive	*N*	113	21%	93	21%	20	20%	0.463
Others	*N*	431	79%	352	79%	79	80%	
Missing		25		18		7		
TBI								
No	*N*	104	19%	70	15%	34	34%	<0.001
Yes	*N*	448	81%	382	85%	66	66%	
Missing	*N*	17		11		6		

A comparison of patients transplanted for VHRR from MSD/MD with those transplanted from MMD revealed no statistically significant difference in patient age, sex-match between donor and recipient, ALL phenotype (T-cell lineage vs. B-cell lineage) and CMV status match. MMD were statistically older compared with MSD/MD. There were more advanced disease, more ex vivo T-cell depletion, and more chemotherapy based conditioning regimen for patients transplanted from MMD as compared to those transplanted from MSD or MD.

An analysis about the global outcomes according to donor type is provided as Supplementary data (Tables S2 and S3).

### Engraftment

Five hundred and forty-one patients achieved neutrophil counts above 0.5 × 10^9^/l at a median time of 20 (range: 0–108) for the whole cohort and 21 (range: 0–78), 18 (range: 6–108) and 25 (range: 12–40) days for BM, PBMC, and CB, respectively. These median times were 21 (0–78) and 25 (12–34), 19 (6–108) and 16 (8–49), and 23 (15–38), and 26 (12–40) days for BM, PBSC and CB for MSD/MD and MMD groups, respectively.

A total of 468 and 426 patients achieved platelet counts above 20 × 10^9^ and 50 × 10^9^/l, respectively. The median time to reach more than 50 × 10^9^/l platelets was 33 (10–286) and 37 (18–54) days, 25 (12–109) and 26 (10–78) days, and 42 (38–67) and 63 (11–106) days for BM, PBMCs and CB for MSD/MD and MMD groups, respectively.

A total of 13 patients died before leukocyte engraftment.

### Graft versus host disease (GvHD)

Three hundred and twenty-three patients did not develop any acute GvHD above grade 1, 126 patients experienced grade 2, 52 grade 3, and 18 grade 4 aGvHD. The cumulative incidences of grade II to IV were 35 and 45% for patients transplanted with BM, 41 and 27% for those transplanted from PBSC and 33% and 53% for those transplanted from cord blood in MSD/MD and MMD group, respectively (*p* = NS for any stem cell sources).

Multivariate analysis revealed that only ex vivo manipulated PB was protective against grade II to IV aGVHD (ex vivo manipulated PBSCs vs. unmanipulated PBSCs: HR 0.17, *p* < 0.001). Neither donor age, gender-match, donor type, remission status, patient age nor TBI showed any statistically significant impact (Supplementary data, Tables S4 and S5).

**Fig. 1:**
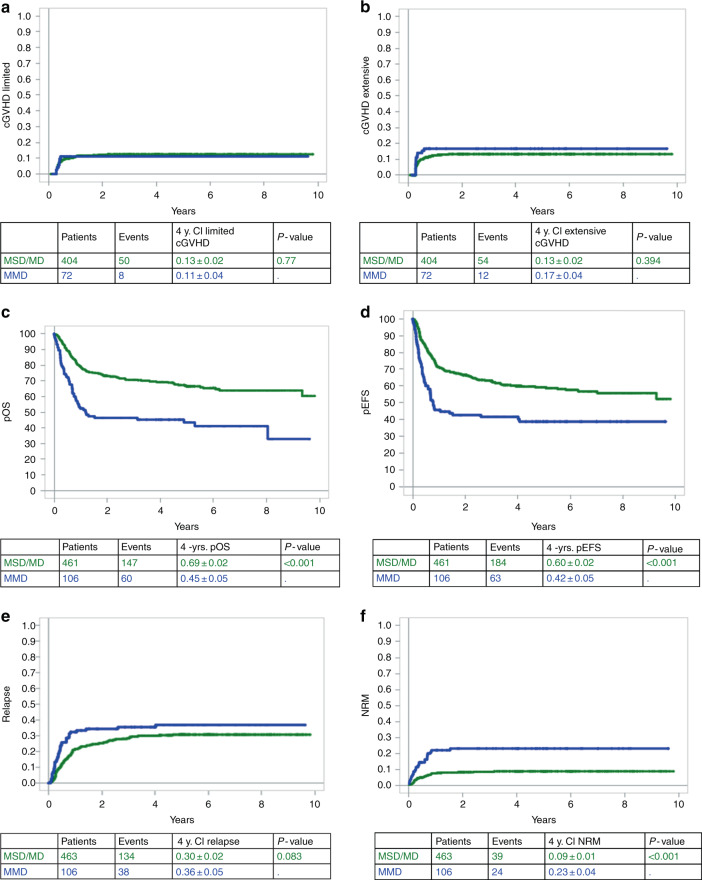
Survival curves at 4 years. 4-year cumulative incidence of: **a** Limited chronic graft versus host disease (GvHD); **b** extensive chronic GvHD; **c** Overall survival; **d** Event-free survival; **e** Relapse; **f** Non-Relapse Mortality. Blue line represents patients transplanted from mismatched donor. Green line: patients transplanted from matched donor.

Among the 476 patients who survived after D100 and were subsequently evaluated for chronic GvHD, 58 and 66 experienced limited and extensive chronic GvHD (cGvHD), respectively. The 4-year CI of limited and extensive cGvHD was 13 ± 2% and 13 ± 2%, respectively for patients transplanted from MSD/MD. These 4-year CI were 11 ± 4% and 17 ± 4% for those transplanted from MMD (Fig. 1a, b). These numbers were not statistically different.

In multivariate analysis, when considering limited and extensive cGvHD altogether, being transplanted from MD represents a significant protective impact factor as compared to MSD (*p* < 0.001), as previously demonstrated by Peters et al. and Balduzzi et al., while there was no difference between MMD and MSD (*p* = 0.2). Unmanipulated BM as compared to unmanipulated PBSCs as well as ex vivo manipulated PBSCs vs. unmanipulated PBSCs are protective against any cGVHD (*p* = 0.009 and *p* = 0.059, respectively). Neither donor or recipient age, gender mismatch, disease status, and TBI in conditioning regimen were significantly associated with any or extensive cGVHD.

### Overall survival

The 4-year overall survival was 65 ± 2% for the entire cohort of 569 patients. Overall survival at 4 years was statistically better for patients transplanted from MSD/MD than for those transplanted from MMD (69 ± 2% vs. 45 ± 5%, *p* < 0.0001) (Fig. 1c). These results were the same when considering separately BFM-2003 and IBFM-2007 studies (Supplementary data, Supplementary Fig. S1A, B). In a multivariate analysis considering donor type, disease status at HSCT, ex vivo graft manipulation, donor-recipient CMV mismatch (i.e., CMV negative donor for CMV positive recipient), patient’s age, TBI in the conditioning regimen and ALL phenotype as risk factors, MMD was associated with a statistically significantly worse OS (*p* = 0.01; HR: 1.94, 95%CI: 1.17–3.21). HSCT for advanced disease (CR > 2 vs. CR1) (*p* < 0.001; HR: 3.36, 95%CI: 2.21–5.11), transplantation from a CMV-negative donor to a CMV-positive patient (*p* = 0.008; HR: 1.59, 95%CI: 1.13–2.23) and transplantation from cord blood vs unmanipulated PBSCs (*p* = 0.012; HR: 2.34, 95%CI: 1.21–4.52) were also negative prognostic factors.

### Event-free survival

The 4-year EFS was 56 ± 2% % for the entire cohort of 569 patients. Fourteen patients developed a secondary malignancies. Among them two had experienced post-HSCT ALL relapse before developing secondary malignancy. The 4-year CI of secondary malignancies was <1%. There was no difference according to donor type (*p* = 0.38). Up to the last follow-up, there were no secondary malignancies reported in the very high risk disease cohort of the International BFM-2007 study.

As for OS, univariate analysis revealed that HSCT from MSD/MD vs. MMD resulted in better outcomes (60 ± 2% vs. 42 ± 5%, *p* < 0.001) (Fig. 1d). The BFM-2003 cohort confirmed such (62 ± 3% vs. 42 ± 6%, *p* < 0.001) but does not exist in IBFM-2007 study (56 ± 4% vs. 48 ± 8%, *p* = 0.14).

In a multivariate analysis adjusted for donor type, disease status at HSCT, ex vivo graft manipulation, donor-recipient CMV mismatch, patient’s age, TBI in the conditioning regimen and ALL phenotype as risk factors, MMD was associated with a statistically significantly worse EFS compared with MSD (*p* = 0.035; HR: 1.67, 95%CI: 1.04–2.68), and MD (*p* = 0.042; HR: 1.52, 95%CI: 1.02–2.26). HSCT for advanced disease (CR > 2 vs. CR1) (*p* < 0.0001; HR: 2.92, 95%CI: 1.98–4.31), transplantation from CB vs. unmanipulated PBSCs (*p* = 0.002; HR: 2.58, 95%CI: 1.40–4.75), and conditioning regimen without TBI (*p* = 0.026; HR: 1.45, 95%CI: 1.05–2.01) were also negative prognostic factors for EFS.

### Relapse

One hundred and seventy two patients relapsed after HSCT, 134 out of 463 transplanted from MSD/MD and 38 out of 106 transplanted from MMD. There was no difference in 4-year CI of relapse between patients transplanted from MSD/MD (30 ± 2%) and those transplanted from MMD (36 ± 5%), *p* = 0.08 (Fig. 1e). However, the difference is statistically significant for patients enrolled in BFM-2003 study (28 ± 3% vs. 39 ± 6%, *p* = 0.02) while the results were superimposable between the two groups in I-BFM-2007 (33 ± 4% vs. 29 ± 8%, *p* = 0.8) (Supplementary data, Fig. S2A, B).

In a multivariate analysis adjusted for donor type, disease status at HSCT, stem cell source, D/R CMV status, TBI in the conditioning regimen, age of patient, and phenotype, disease status (CR2 vs. CR1 and CR > 2 vs. CR1; *p* = 0.006, HR: 1.74, 95%CI: 1.18–2.57 and *p* < 0.001, HR: 2.59, 95%CI: 1.54–4.35), stem cell source (cord blood vs. unmanipulated PBSCs and ex vivo manipulated PBSCs vs. unmanipulated PBSCs; *p* = 0.001, HR: 3.31, 95%CI: 1.60–6.87 and *p* = 0.012, HR: 2.37, 95%CI: 1.21–4.63) and conditioning regimen w/o TBI (*p* = 0.043, HR: 1.50, 95%CI: 1.01–2.21) appeared to be statistically significant negative prognostic factors for relapse.

### Non-relapse mortality (NRM)

The 4-year NRM was 11 ± 1% in the whole cohort. The 4-year NRM was lower for patients transplanted from MSD/MD (9 ± 1%) than from MMD (23 ± 4%), *p* < 0.001 when considering both studies (Fig. 1f). These results were confirmed when the BFM 2003 and I-BFM 2007 studies were considered separately (data not shown). The multivariate analysis adjusted for donor type, disease status at HSCT, stem cell source, donor-recipient CMV status, TBI in conditioning regimen and age of patient showed that MMD was associated with a higher NRM in comparison to MSD (*p* = 0.031; HR: 2.62, 95%CI: 1.09–6.25) as well as disease status (CR > 2 vs. CR1; *p* = 0.012; HR: 2.36, 95%CI: 1.21–4.60) and CMV status (D−/R+ vs. others; *p* < 0.001; HR: 3.02, 95%CI: 1.70–5.34).

## Discussion

Allogeneic HSCT still remains one of the most effective available treatments for pediatric patients suffering from poor-risk malignant hematological diseases, particularly for those with poor-risk acute lymphoblastic leukemia [5, 11–17]. For decades, both conventional chemotherapy and HSCT have augmented the potential of cure, particularly after MSD- and MD-HSCT [18–22]. HSCT from the so-called “alternative” donor, i.e., mismatched cord-blood, <9/10 (un)related-donor, T-cell depleted haplo-identical graft, and more recently posttransplant cyclophosphamide T-repleted haplo-identical transplantation remain more questionable. Indeed, HSCT from such alternative donors—whether related or unrelated—remained associated with high treatment failure rates, reflecting higher non-relapse mortality as compared to HSCT from HLA-compatible donors and is therefore often limited to high risk patients and to centers which perform ex vivo graft manipulations, or partially HLA-matched unrelated cord blood transplantation [14, 21, 23–25].

In 2003, the Austrian–German–Swiss BFM Study Group initiated a prospective study to evaluate the feasibility of the systematic use of either 9 or 10/10 unrelated donors for patients <18 years with an indication of allogeneic HSCT for ALL in first or subsequent CR. In 2007, the International-BFM consortium reproduced and extended the same study design into the International BFM 2007 study to ten countries (Czech Republic, Denmark, France, Israel, Italy, The Netherlands, Poland, Sweden, Slovakia, Turkey). In both studies, patients were allocated to different risk groups based on their relapse risk. Patients with standard risk disease in frontline were not eligible for HSCT. HRR-patients were stratified to HSCT exclusively from MD or MSD, if available, while only patients with VHRR could undergo HSCT from MMD, in case of a MSD or MD donor was lacking.

Both studies demonstrated the equivalence between MSD and MD, regardless of the relapse risk. The 4-year OS was 79 ± 4% and 73 ± 3% for patients transplanted from MSD and MD, respectively (*p* = NS) in the BFM-2003 study and 72 ± 4% and 68 ± 4% (*p* = NS) in the IBFM-2007 study. 4-year EFS and CIR were also similar in both groups. NRM was lower in the MSD-group in the BFM-2003 study but appeared similar between MSD and MD-groups in the I-BFM-2007 [5, 6]. Because of these published results, we decided to merge MSD and MD transplanted patient sub-groups for analysis.

Out of the 148 patients transplanted from MMD, 106 had VHRR and had an indication for a MMD graft according to protocol where 42 patients with HRR were transplanted from MMD by center’s decision. The overall results were inferior to those obtained with better-matched donors with 4-year OS and EFS of 56 ± 4% and 52 ± 4%, respectively. In contrast, the results were significantly better for the 42 HRR-patients transplanted from MMD with 4-year OS and EFS of 82 ± 6% and 80 ± 6%, respectively [7].

The aim of this paper is to assess the outcome of the VHRR patients enrolled in both BFM ALL SCT 2003 and I-BFM-ALL SCT 2007 according to the donor type.

Here we demonstrated a better overall result for these VHRR patients transplanted from well-matched donors, defined as >9/10 related or unrelated donors or ≥5/6 matched cord-blood as compared to those grafted with a so-called alternative graft. The number of patients in some sub-groups as unrelated cord-blood or PBSC with different kind of ex vivo T-cell depletion did not allow to analyze these sub-groups separately. In multivariate analyses, the MMD type consistently appeared as a negative prognostic factor for OS, EFS, NRM, and CI of extensive cGvHD. Moreover, even though alternative donor-HSCT did not have negative impact on CI of relapse, there was no positive-impact for event-free survival, i.e., the putative stronger graft-versus-leukemia effect conferred by the higher mismatch-level did not appear. To note, there were no post-transplant cyclophosphamide T-cell repleted HSCT in these studies.

In the present paper, relapse was the main cause of failure, higher than NRM, consistent with the literature, with a 4-year CIR about 30–35%. In contrast, Peters et al. reported a CIR from of 22–24% in patients transplanted from MSD or MD [5]. Mo et al. reported similar results for HSCT from haplo-identical donor or unrelated cord blood [26]. Michel et al. reported as well 14.9–23.4% CI of relapse in the French randomized trial comparing transplantation from either one or two cord blood unit in patients below 35 years with leukemia [27]. This latter study was exclusively dedicated to cord blood transplantation. Bertaina et al. reported a retrospective multicentric Italian study comparing the results obtained in pediatric population transplanted from either MUD, MMUD, and haplo-identical αβ-T cell and B cell depleted transplantation for ALL or AML in complete remission. In this paper, haplo-identical αβ-T-cell and B cell depletion provided the same GvHD-relapse-free survival than HSCT from MUD that appeared statistically better than HSCT from MMUD [28]. In the current report, being transplanted from cord blood compared with other stem cell sources led to worse results due to higher relapse incidence and lower OS and EFS rate. However, the majority of published papers reported results for a mixture of HRR and VHRR patients while this analysis evaluates the outcome of worst patient-group only. Nevertheless, all these studies demonstrated the need for developing new approaches in order to increase the cure rate and to decrease the relapse incidence. Our algorithm of risk-group allocation was based on a combination of both biological and clinical criteria at time of disease occurrence and MRD level at the end of induction and of consolidation courses, in first and second line therapy [2, 6, 29]. Obtaining a better disease control before HSCT represents the absolute challenge we have to face [30, 31]. Better disease control means low or negative stable MRD level reaching the concept of low tumor burden developed in solid tumor setting. Having solely one low MRD time-point is probably not sufficient since this kind of result may be only “cosmetics” and followed by rapid disease re-appearance. New immonological approaches might be an alternative to toxic chemotherapy. Monoclonal antibodies either combined with chemotherapy as inotuzumab (directed against CD22) or bi-specific like BiTe-blinatumomab (directed against CD19) currently demonstrated feasibility and efficacy in Phase II-studies [32–36]. For example, Von Stackelberg et al. published the results of a phase I/II study of blinatumomab in children. In this paper, among 70 patients below 18 year of age who received the recommended dosage (i.e., 15 μg/m^2^/d × 21 days), 27 (39%) obtained morphological complete remission and 14 MRD complete remission [37]. Another promising approach is the application of gene-modified T-cells—at least in some countries and some centers. Some of these CAR-T cells are now available on the market and their effectivity should be evaluated in prospective trials [38, 39]. Although treatment-related mortality is low in the short-term-observation, the long-term side effects of complete B-cell aplasia in young patients has to be followed carefully. Another concern about these immunotherapies is the risk of relapse especially from CD19 or CD22 negative B-cell clones [40–44]. However whether some patients will be cured with conventional chemotherapy and immunotherapy without HSCT or if the cell therapy is a bridge to allogeneic HSCT has to be evaluated in prospective trials. To date, allogeneic HSCT is still the most efficient immunotherapy. The impact of pre- and post-HSCT MRD levels are well described [16, 21, 24, 25]. The lack of consistent collection of MRD level in both protocols BFM-2003 and IBFM-2007 represents probably the most important limitation of this paper. Indeed, we may speculate that some patient sub-groups, according to MRD, may benefit of HSCT from alternative donors where others have to be avoided due to unsatisfying benefit-risk balance and might be allocated to other therapies.

To decrease NRM, reduction of early and late transplant associated toxicity appears as crucial [45, 46]. Beside immunotherapeutic approaches, the development of reduced-toxicity conditioning regimen and substitution of TBI- should allow to decrease NRM. A prospective randomized trial is currently evaluating this option (Eudract N° 2012-003032-22, see also www.clinicaltrials.gov). A recent retrospective study from Pediatric Diseases Working Party of the European society for Blood and Marrow Transplantation (PDWP-EBMT) with more than 1400 pediatric patients transplanted for ALL have demonstrated the superiority of TBI-based conditioning regimen, especially in CR2-patients [47].

In the present study, we demonstrated the feasibility of allogeneic HSCT for very-high risk of relapse patients from either MSD/MD or MMD donor with inferior overall results for the latter. However, using MMD-HSCT for HRR-patients offer the same chance of success as grafts from HLA-compatible donors. However, further progress is needed to decrease overall treatment failure, i.e., both relapse rate and treatment-related mortality in VHRR patients.

## Supplementary information

Supplementary data

Supplementary Tables S1–S5

Supplementary Figs. S1, S2
