# Supplementary material for: The impact of donor type on the outcome of pediatric patients with very high risk acute lymphoblastic leukemia. A study of the ALL SCT 2003 BFM-SG and 2007-BFM-International SG
Source: Bone Marrow Transplant. 2020 Aug 4;56(1):257–66. doi: 10.1038/s41409-020-01014-x (PMC7796856; doi:10.1038/s41409-020-01014-x)
Supplement: Supplementary file 1 — Supplementary data [file 41409_2020_1014_MOESM1_ESM.docx]

Supplemental data

CS1 - Patients and Methods

As already published, transplant centers from 3 countries (Austria, Germany, and Switzerland) participated in the BFM ALL SCT 2003 study from 2003 to 2011, and 10 additional countries (Czech Republic, Denmark, France, Israel, Italy, the Netherlands, Poland, Sweden, Slovakia, and Turkey) participated in the International BFM ALL SCT 2007 study from 2007 to 2011. Both studies were prospective, multicenter open trials (extended as a register studies until 2013), approved by the central and local ethical committees. Informed consents were obtained from parents or legal guardians and assent from patients, when appropriate, prior to study entry.

*Inclusion Criteria*. All consecutive patients up to the age of 18 years at the time of initial ALL diagnosis or relapse, with an indication for allogeneic HSCT, according to national frontline and relapse protocols, were eligible. Complete remission was defined based on bone marrow (BM) with active hematopoiesis and fewer than 5% leukemic blast cells (identified morphologically) and normal cerebrospinal fluid.

*Donor type*. HLA-mismatched donors (MMDs) were defined as donors with more than one allelic or antigenic disparities up to a different haplotype (MMD), regardless of their relationship with the recipients meaning they may be related or unrelated. Ex-vivo either positive CD34+ or negative CD3/CD19 selection was accepted at center’s discretion. There was no CD3-TCR αβ-depletion in these studies. Related and unrelated fully or partially HLA-matched cord blood (CB) was also accepted as a stem cell source. For cord blood, HLA typing was defined using low-resolution molecular techniques for HLA-A and HLA-B and high resolution typing for HLA-DR, and any cord blood unit with less than 5/6 matches with the recipient were classified as MMD.

*Risk stratification*. The patients were stratified according to BFM eligibility criteria for transplantation: standard relapse risk (SRR) patients were not eligible for any HSCT, high relapse risk (HRR) patients were eligible for either MSD or MD HSCT, and very high relapse risk (VHRR) patients also had indication for MMD transplants.

Indications for allogeneic HSCT according to BFM-frontline protocols

Risk definition and indications for allogeneic hematopoietic stem cell transplantation were summarized in both supplemental table 1a and 1b for patients in CR1 and CR2, respectively. Briefly, stratification was based on prednisone response, some fusion-transcripts or gene abnormalities and MRD level at day 78 in CR1. MRD may be evaluated locally either by flow-cytometry or molecular technics as center discretion. MRD data were not registered in the database. Any-T-ALL relapse and early and very early B-ALL relapses as well as high MRD-levels after reinduction-chemotherapy were considered as eligibility criteria for patients in CR2, where “high MRD levels” were not defined but mainly above 10^-4^.

The closest MRD level to transplantation as well as MRD level post transplantation were not mandatory and therefore not consistently registered.

*Stem cell source*. Bone marrow (BM) was the recommended source according to the protocol, but granulocyte colony-stimulating factor-primed peripheral blood (PB) and cord blood (CB) stem cells were also acceptable sources, according to transplant or donor center preference. Target doses of >3x10^8^ nucleated cells (NC)/kg recipient body weight and >1.5x10^6^ CD34^+^ cells/kg recipient body were recommended for both BM and PBSC. For CB, the target doses were 3x10^7^ nucleated cells (NC)/kg recipient body weight and >1x10^6^ CD34^+^ cells/kg recipient body.

*Transplant procedure*. The myeloablative conditioning regimen was based on both recipient age and donor type and did not depend on disease risk. For patients above 2 years of age and transplanted from either MSD or MD for VHRR disease, the conditioning regimen was based on hyper-fractionated total body irradiation (TBI, dose 1200, 200 cGy bid on days -7 to -5, and etoposide (60 mg/kg at D-3). Patients 2 years or younger received body-weight adjusted doses of busulfan (either I.v. or p.o. with or without PK sampling and adjustment, depending the center) from D-11 to D-8, followed by etoposide (40 mg/m^2^ at D-4) and cyclophosphamide (60 mg/kg for 2 days, D-3 and -2). For patients transplanted from MMD for VHRR disease and >2 years the conditioning regimen was based on hyper-fractionated total body irradiation (TBI, dose 1200, 200 cGy bid on days -10 to -8), fludarabine (40 mg/m^2^/d for 4 days from D-7 to D-4) and etoposide (40 mg/kg at D-3). Patients 2 years or younger received body-weight adjusted doses of either IV or oral busulfan from D-11 to D-8, followed by fludarabine (40 mg/m^2^/d from D-7 to D-4) and cyclophosphamide (60 mg/kg for 2 days, D-3 and -2).

GVHD prophylaxis was cyclosporine-A based, alone for those patients transplanted from MSD and associated with short-methotrexate (D+1, D+3 and D+6) and anti-thymocyte globulin (ATG Fresenius 20 mg/kg/dose, on days -4, -3, -2) for patients transplanted from MD. Methotrexate was substituted with steroids in CB recipients. GvHD prophylaxis for patients transplanted with ex-vivo T-cell depleted graft depended on both the center and the amount of residual CD3^+^ cells into the cell therapy product.

Neutrophil engraftment was defined as the first of 3 consecutive days with more than 0.5 G/l neutrophils. Platelet engraftment was defined as the first of 7 days with more than either 20 or 50 G/l platelets without any transfusion support.

Acute and chronic GVHD were graded as previously described. i.e. according to modified Glucksberg criteria for acute GvHD and to ancient NIH criteria for chronic GvHD (meaning limited or extensive). Patients who were alive and in remission 100 days after HSCT were considered at risk for chronic GVHD. The discontinuation of immunosuppression with no clinical signs of GVHD was considered as the absence of GVHD.
