# Supplementary material for: The impact of donor type on the outcome of pediatric patients with very high risk acute lymphoblastic leukemia. A study of the ALL SCT 2003 BFM-SG and 2007-BFM-International SG
Source: Bone Marrow Transplant. 2020 Aug 4;56(1):257–66. doi: 10.1038/s41409-020-01014-x (PMC7796856; doi:10.1038/s41409-020-01014-x)
Supplement: Supplementary file 2 — Supplementary Tables S1–S5 [file 41409_2020_1014_MOESM2_ESM.docx]

**Supplemental Table S1: Risk definition and indications for allogeneic HSCT in ALL according to the BFM criteria**

1. **Patients in CR1**

|  | | PCR-MRD results | | | | |
| --- | --- | --- | --- | --- | --- | --- |
|  | | MRD-SR | MRD-MR | MRD-HR | | no MRD result |
|  |  |  |  | MRD-TP2 ≥10^-3^ | MRD-TP2 ≥10^-2^ |  |
| HR criteria (in hierarchical order) | No CR d33 | **n.a.** | **VHRR** | **VHRR** | **VHRR** | **VHRR** |
|  | PPR + (9;22) | **VHRR** | **VHRR** | **VHRR** | **VHRR** | **VHRR** |
|  | PPR + (4;11) | HRR | HRR | HRR | **VHRR** | HRR |
|  | PGR + (9;22) | *no* | HRR | HRR | **VHRR** | HRR |
|  | PGR + MLL | *SHR* | *SHR* | HRR | **VHRR** | HRR |
|  | PPR +* | *no* | *no* | HRR | **VHRR** | HRR |
|  | PPR only§ | *no* | *no* | HRR | **VHRR** | HRR |

**VHRR** (very high relapse risk): MMD (mismatched donor) indicated, if no MSD and no MD available

**HRR** (high relapse risk): MD (matched donor) donor indicated, if no MSD available

***SHR*** *(standard high risk)*: MSD (matched sibling donor) only indicated

***no*** = no HSCT indicated

* PPR (prednisone poor response) + pre-B ALL or T-ALL and/or more than 1000 blasts on d15 after chemotherapy start and/or initial WBC>100,000/µl

§ PPR only

MRD-SR (minimal residual disease – standard risk): MRD negativity after 4 and 12 weeks induction treatment, measured with two independent MRD targets with a sensitivity of ≤10^-4^.

MRD-MR (minimal residual disease-medium risk): any MRD positivity after 4 and 12 weeks induction treatment, but <10^-3^ at week 12.MRD-HR (minimal residual disease-high risk: MRD ≥10^-3^ at week 12.

In addition, children below 6 months of age at diagnosis and a white blood cell count > 300.000/µl and MLL-rearrangement were considered for very high relapse risk.

**B. Patients after first relapse**

| - T-lineage: any BM involvement  - BCP-ALL: very early BM involving relapse, early isolated BM relapse  - >CR 2: according to risk for TRM | **VHRR** |
| --- | --- |
| - MRD * > 10^-3^:  - B-cell precursor:  early combined BM relapse,  late BM relapse | **HRR** |
| Intermediate risk:  - MRD * < 10^-3^:  - B-cell precursor:  : early combined BM relapse,  late BM relapse | ***SHR*** |

* MRD detected after the second induction block

**VHRR**: mismatched donor indicated, if no MSD and no MD available

**HRR:** matched donor indicated, if no MSD available

***SHR*:** only matched sibling donor indicated

**Timepoint of relapse:**

very early: <18 months after primary diagnosis

early: > 18 months after primary diagnosis and < 6 months after cessation of front-line therapy

late: > 6 months after cessation of front-line therapy

**Supplemental Table S2: Outcome of subgroups by donor type**

**Supplemental Table S3. Outcome: multivariate analyses**

**Supplemental Table S4. GVHD according to stem cell source and donor type**

**Supplemental Table S5. Probability of acute graft-versus-host disease: multivariate analyses**
