# Supplementary material for: The impact of donor type on the outcome of pediatric patients with very high risk acute lymphoblastic leukemia. A study of the ALL SCT 2003 BFM-SG and 2007-BFM-International SG
Source: Bone Marrow Transplant. 2020 Aug 4;56(1):257–66. doi: 10.1038/s41409-020-01014-x (PMC7796856; doi:10.1038/s41409-020-01014-x)
Supplement: Supplementary file 3 — Supplementary Figs. S1, S2 [file 41409_2020_1014_MOESM3_ESM.docx]

Supplemental Figure S1

A B


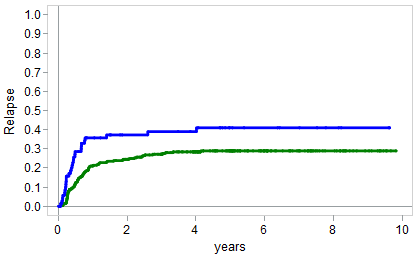

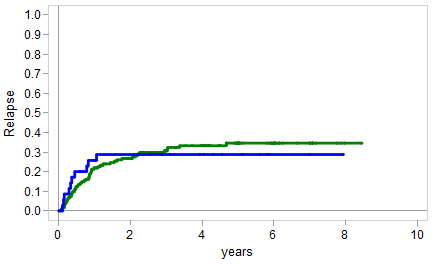

4-year cumulative incidence of relapse for A. patients transplanted on ALL-SCT BFM 2003 study; B. patients transplanted on ALL-SCT IBFM 2007 study.

Blue line represents patients transplanted from mismatched donor. Green line: patients transplanted from matched donor

Supplemental Figure S2

A B


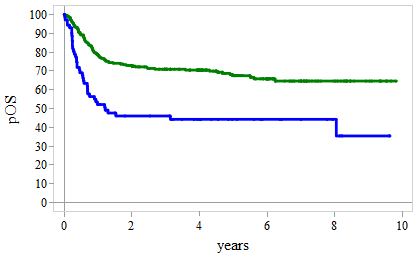

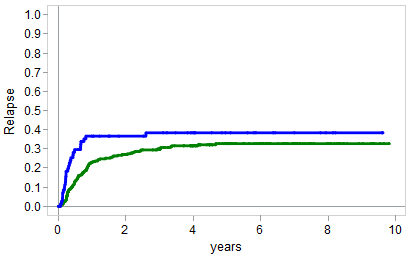


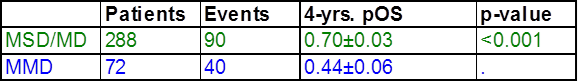

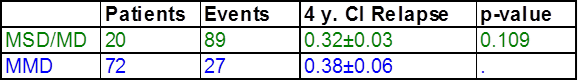


C D


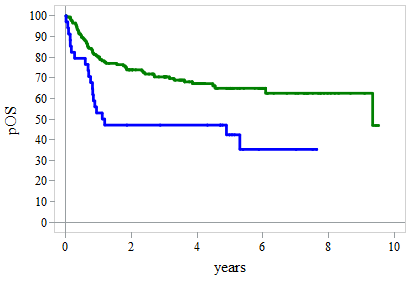

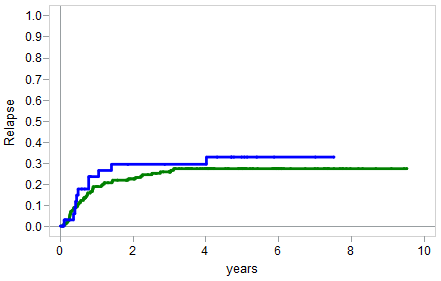


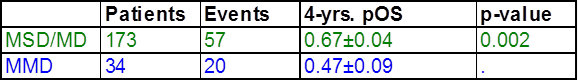


4-year overall survival and cumulative incidence of relapse for: A and B, patients below or equal 12 years; C and D, patients above 12 years. Blue line represents patients transplanted from mismatched donor. Green line: patients transplanted from matched donor
